# Light-Sensing Protein Illuminates Sun-Loving Ocean Bacteria

**DOI:** 10.1371/journal.pbio.0030287

**Published:** 2005-07-19

Biologists have a knack for finding life in the harshest or loneliest regions of the Earth. Microbes, in particular, have adapted to wild variations in environment, and different strains of bacteria thrive under extremely acidic, sweltering, low-nutrient, and even oxygen-free conditions. Unfortunately, humans lack such flexibility, so they cannot always create lab conditions cozy enough for both biologists and their bacterial cultures. Until recently, no bacterial culture meant no genomic information.

Enter the polymerase chain reaction, a technique to amplify genes, and the environmental shotgun sequencing approach, a strategy to order vast numbers of genes. With the advent of these techniques, harvesting precious bacterial cultures was no longer the sole strategy for understanding bacterial genetics. Like the earlier shotgun approach, which compiles sequences of randomly fractured human genes, the environmental shotgun approach compiles sequences of bacterial genes skimmed from the top layer of the ocean. These bacteria are known as oligotrophs; given the dearth of nutrients in their environment, they must make use of a variety of energy sources. Findings from meta-genetic studies of Sargasso Sea bacteria corresponded with earlier discoveries of proteorhodopsin, a membrane protein that harnesses sunlight's energy. Scientists needed to do further research to understand how many and which marine bacteria had these light-sensitive proton-pump proteins.

In a new study by scientists from Israel, Austria, Korea, and the United States, Oded Béjà and colleagues sought answers to these questions by isolating large segments of DNA from the top, or photic layer, of the Mediterranean and Red Seas. Then, they inserted large segments of this DNA into host bacteria. This creates what is known as a large-insert bacterial artificial chromosome library, an amplified collection of the genome of interest. Like shotgun sequencing, this technique helped solve the human genome and now is helping to solve more exotic genomes.

By analyzing the library, the scientists located specific genes in relation to the entire genome. This analysis led to several insights. By estimating the average size of bacterial genomes, Béjà et. al were able to calculate that 13% of bacteria near the ocean's surface contain proteorhodopsin. While their bacterial artificial chromosome library revealed diversity among proteorhodopsin genes, it also revealed that proteorhodopsin is uniquely suited to make use of the high-radiation sunlight that illuminates the sea. Some evidence suggests that many of the bacteria with proteorhodopsin might also be able to metabolize sulfur, a common energy source for deep sea life. Additionally, Béjà and colleagues found some potential evidence that the marine bacteria are able to manufacture retinal, a molecule typically associated with vision.

If we can believe that austere, solitary stretches of open ocean are in reality rife with diverse bacteria, it's no great stretch to imagine that these bacteria make use of light energy. What else would they feed on in the nutrient-poor upper layers of foam and froth? Many other organisms use proteins resembling proteorhodopsin for different functions. Humans, for instance, use rhodopsin to sense light in the eyeball. The presence of rhodopsin-like proteins in a wide range of life may eventually provide hints to the protein's evolutionary age. That this large class of transmembrane proteins was so well-conserved over a long evolutionary time scale provides evidence for complex ancient proteins. Another question that remains is whether the proteorhodopsin has any sensory function as does rhodopsin in humans, or whether the bacteria use the protein purely for energy transduction. [Fig pbio-0030287-g001]

**Figure pbio-0030287-g001:**
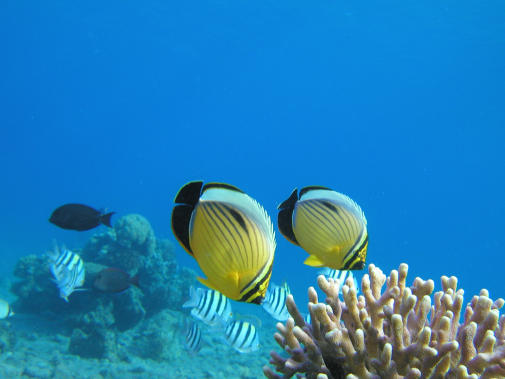
Ocean bacteria with the light-sensitive proteorhodopsin enzyme live several meters above this coral reef (Photo: Boaz Harel)

